# Supplementary material for: A Theoretical and Practical Analysis of Membrane Protein Genes Altered in Neutrophils in Parkinson’s Disease
Source: Curr Issues Mol Biol. 2025 Jun 13;47(6):459. doi: 10.3390/cimb47060459 (PMC12191767; doi:10.3390/cimb47060459)

Supplementary information for**: A theoretical and practical analysis of membrane protein genes altered in neutrophils in Parkinson's Disease**

ITEM	PAGE
**Supplemental Table S1.** Primers receptors of basophils, monocytes and neutrophils.	**2**
**Supplemental Table S2.** Sequences of primers used for dPCR assay.	**3**
**Supplemental Table S3**. Data used for the elaboration of graph 1A, data obtained from the GSE99039 database.	**4**
**Supplemental Table S4.** Data used for the elaboration of graph 1B, data obtained from the GSE99039 database.	**16**
**Supplemental Table S5.** Data used for the elaboration of figure 2, data obtained from SHINY software.	**28**
**Supplementary Figure S1**. End point PCR, evaluation of neutrophil enrichment through 1: Non Template Control (NTC), 2: Positive Control, 3: Healthy control, 4: Parkinson Disease.	**34**

Supplemental Table S1. Primers for End Point PCR to find receptors of basophils, monocytes and neutrophils.

Primers name	Sequence	Cycling parameters
**CXCR2-F**	CAAGGTGGTCTCACTCCTG	95°C for 10 min and 45 cycles; 95°C for 10 s; Gradient 57°C-54ºC for 33 s; 72°C for 17 s
**CXCR2-R**	GTATTGTTGCCCATGTCCTC	
**FCGR3B-F**	GGTACAGCGTGCTTGAGAAG	
**FCGR3B-R**	GCACCTGTACTCTCCACTGT	
**FCGR2B-F**	CTGTGCATCTGACTGTGCT	
**FCGR2B-R**	AGCCTATGTTTCCTGTGCAG	
**PRG2-F**	CCTCCTGTTTACTTGCCGGA	
**PRG2-R**	AGACTTGACCCTGGTTGAGC	
**FCGR3A-F**	CCAGACAAACCTCTCCACC	
**FCGR3A-R**	AAATACTTCCTGCCTTTGCCA	

Supplemental Table S2. Sequences of primers used for dPCR assay. All primers were acquired from Oligo T4 (Mexico). The primers were divided into groups according to the annealing temperature and time.

Primers receptors

**Grupo**	**Primers name**	**Sequence**	**Cycling parameters**
**1**	P2RX1-F	TGCATATGGAGCTCTGTGCC	95°C for 5 min and 44 cycles; 95°C for 15 s; 58°C for 31 s; 60°C for 15 s.
	P2RX1-R	GAGTCACTGTGAGTGGACAAGA	
	ORAI3-F	AGCTGTGAGCAACATCCACA	
	ORAI3-R	GGGCACAAACTTGACCCAAC	
	TLR1-F	TGTCCAGAGTGAATGGTGCC	
	TLR1-R	GTACTGCGGAATGGGTTCCA	
	TLR2-F	CTCCTTCACTCAGGAGCAGC	
	TLR2-R	GCTGTCCTGTGACATTCCGA	
**2**	CLCN2-F	GTGAAGGATATTGACCCGGA	95°C for 5 min and 44 cycles; 95°C for 15 s; 55°C for 34 s; 60°C for 15 min.
	CLCN2-R	GGTTGTATTCACCATCATCCAG	
	KCNE4-F	GCAACGAGTACTTCTACATTCTG	
	KCNE4-R	TCCTCGTCTTTGTACAGCAG	
	SNCA-F	GGGTGTTCTCTATGTAGGCTC	
	SNCA-R	TTAGGCTTCAGGTTCGTAGTC	
**3**	CACNG8-F	GCGTGAAGATCAATCATTTCCC	95°C for 5 min and 44 cycles; 95°C for 15 s; 56°C for 30 s; 60°C for 1 min.
	CACNG8-R	GAGCGTGTTGGTGTTGGA	
	KCNJ15-F	GTCCTCATGCCATCTTCCT	
	KCNJ15-R	GTCCTCAGTTCTCTTTCCCTC	
	ACTIN-F	AAGAGAGGCATCCTCACCCT	
	ACTIN-R	TACATGGCTGGGGTGTTGAA	

Supplemental Table S3. Data used for the elaboration of graph 1A, data obtained from the GSE99039 database.

	ID GEN	Log 2 Fold Change	Log 10 *P*-value
**Ion Channels**	1555246_a_at	7.35225263	0.0904
	210383_at	4.96578428	0.717
	205508_at	-5.2598064	0.279
	229057_at	6.82173792	0.209
	206381_at	5.41734766	0.377
	235225_at	6.41734766	0.204
	210364_at	5.20556334	0.399
	210363_s_at	4.53616832	0.833
	210363_s_at	4.53616832	0.833
	232512_at	-7.3928946	0.0726
	204722_at	-4.7027499	0.545
	204723_at	-6.148161	0.228
	206981_at	6.41734766	0.173
	236359_at	-6.0023102	0.373
	207413_s_at	5.26534457	0.363
	207413_s_at	5.26534457	0.363
	228504_at	5.71028355	0.478
	244708_at	5.94786238	0.307
	229199_at	4.65108776	1.74
	206950_at	5.53951953	0.764
	208578_at	5.27090409	0.41
	210853_at	5.09295553	0.393
	220791_x_at	5.84476888	0.326
	220791_x_at	5.84476888	0.326
	208437_at	-11.565246	0.00349
	213499_at	3.29335894	2.73
	201733_at	3.66383089	0.996
	201735_s_at	-4.4739312	0.411
	201734_at	-4.5496201	0.289
	201732_s_at	5.13796526	0.284
	205148_s_at	-2.6438562	2.95
	205149_s_at	-3.9885044	1.48
	214769_at	-3.8180706	0.996
	217556_at	-5.2378638	0.629
	231066_s_at	-11.650798	0.00349
	232127_at	3.90400809	0.873
	226273_at	4.46434514	0.747

	206704_at	6.78627323	0.175
	232128_s_at	8.05505162	0.057
	226274_at	8.09788782	0.0386
	203950_s_at	-5.5195281	0.352
	221961_at	4.1896803	0.757
	38069_at	5.0540927	0.588
	209235_at	-4.8119789	0.455
	1554748_at	4.08314124	1.08
	1554749_s_at	-8.0359933	0.0472
	205985_x_at	8.17918792	0.0334
	210403_s_at	-5.7178568	0.324
	210402_at	-6.5872727	0.144
	206765_at	-2.8889687	1.52
	233059_at	5.17397021	0.614
	207141_s_at	-4.8282808	0.578
	207142_at	5.65108776	0.42
	211451_s_at	3.55300276	1.31
	208359_s_at	10.6186856	0.00789
	211304_x_at	3.18442457	2.2
	208397_x_at	4.94786238	0.521
	238780_s_at	-5.5597919	0.282
	211817_s_at	-5.7485536	0.248
	237186_at	-6.3335161	0.161
	208404_x_at	-7.8152246	0.0545
	232411_at	5.20024954	0.903
	232411_at	5.20024954	0.903
	205304_s_at	-5.4297314	0.402
	205303_at	-6.2653446	0.21
	244113_at	4.44850859	0.896
	207527_at	5.82828076	0.296
	228581_at	4.36289988	1.23
	206692_at	-5.2933589	0.399
	231740_at	5.29335894	0.437
	210179_at	4.50307753	1.01
	211427_s_at	6.23242994	0.389
	220776_at	5.73312353	0.228
	211806_s_at	-2.4579896	2.92
	210119_at	-2.6170561	2.26
	238428_at	-2.9323613	2.03
	230585_at	-2.3437325	1.83
	239464_at	-4.7216583	0.35

	219564_at	5.79585928	0.364
	222901_s_at	-8.1750122	0.0506
	221307_at	6.09788782	0.195
	1555230_a_at	3.33642766	2.57
	224528_s_at	5.50635267	0.496
	221321_s_at	6.03032454	0.223
	223727_at	-7.4447335	0.0711
	231774_at	3.45640514	1.64
	228269_x_at	5.55979192	0.308
	1569355_at	6.13796526	0.234
	1555694_a_at	-5.9657843	0.23
	233688_at	6.34519787	0.226
	224530_s_at	-5.5803532	0.367
	236783_at	-6.9284021	0.103
	210263_at	3.56149422	1.32
	236407_at	-3.7858752	1.7
	208514_at	5.27090409	0.343
	221095_s_at	5.0540927	0.516
	222922_at	-2.7661119	1.84
	227647_at	-3.3219281	1.49
	222923_s_at	-3.7350433	0.924
	222379_at	4.07096652	1.88
	1552507_at	3.77004299	1.81
	1552508_at	4.46434514	1.05
	220010_at	-5.0493076	0.545
	206842_at	-4.2002495	1.3
	207103_at	-5.3393451	0.703
	213832_at	4.56661319	0.967
	211301_at	5.12784104	0.438
	215014_at	6.42973138	0.298
	211827_s_at	6.67300254	0.18
	208477_at	-5.1844246	0.611
	230547_at	-7.133907	0.136
	222289_at	3.53115606	2.09
	240614_at	4.83238516	0.652
	207600_at	4.95232202	0.472
	230531_at	-4.9657843	0.393
	243893_at	-11.480357	0.0048
	208251_at	4.94786238	0.547
	228436_at	6.64963854	0.141
	235467_s_at	-7.2944909	0.107

	207162_s_at	3.38232537	1.48
	235781_at	-5.9213902	0.246
	208020_s_at	4.36289988	0.9
	211592_s_at	5.49980982	0.6
	238636_at	5.18442457	0.434
	242973_at	5.76415042	0.321
	243334_at	-3.1584294	3.22
	1555993_at	-4.1844246	1.25
	210108_at	6.07825901	0.335
	207998_s_at	-7.3953214	0.0701
	208432_s_at	3.14560532	2.97
	236013_at	-4.1379653	0.963
	244256_at	4.86564761	0.719
	238747_at	5.9567955	0.217
	240650_at	-8.4397155	0.0306
	242410_s_at	-10.431723	0.0132
	208377_s_at	-8.5221776	0.0264
	211802_x_at	3.83856373	1.46
	211314_at	3.91267295	1.4
	211315_s_at	4.36289988	1.2
	210380_s_at	6.79265685	0.137
	207869_s_at	9.30585973	0.032
	222960_at	3.3075728	2.02
	205845_at	-5.6012119	0.243
	208299_at	3.13289427	1.98
	211830_s_at	3.74661576	1.64
	221631_at	3.82623293	1.2
	217515_s_at	3.73312353	1.41
	206996_x_at	-3.917025	1.39
	210967_x_at	-4.7331235	0.58
	210185_at	5.54624539	0.255
	213714_at	4.84892053	0.955
	215365_at	4.83238516	0.785
	207776_s_at	6.32192809	0.202
	1555098_a_at	-6.9407555	0.138
	1559420_x_at	-7.7239441	0.0472
	1559419_at	-8.9372151	0.0195
	209530_at	-5.9839316	0.223
	34726_at	7.58450091	0.0862
	207693_at	-3.5977144	1.18
	243244_at	6.33351607	0.221

	206612_at	5.96578428	0.218
	214495_at	3.6384562	1.72
	244099_at	4.16617886	1.13
	206384_at	-4.8698599	0.636
	62987_r_at	5.71785677	0.238
	231737_at	-6.4932965	0.207
	221585_at	-7.011588	0.108
	221401_at	-5.5872727	0.411
	1552602_at	-6.078259	0.298
	224291_at	3.89969509	1.72
	1552863_a_at	-4.3898669	0.65
	224137_at	4.0540927	1.75
	234756_at	3.36141638	2.04
	234750_at	3.55131045	1.9
	231355_at	10.5674339	0.00656
	230849_at	4.72165834	0.917
	208479_at	6.75789143	0.114
	208479_at	6.75789143	0.114
	239118_at	-6.5195281	0.211
	207237_at	2.94341647	1.52
	207248_at	5.87832144	0.333
	206762_at	6.07825901	0.181
	1553347_s_at	6.41734766	0.17
	232936_at	3.77004299	2.34
	208560_at	4.82828076	0.648
	210078_s_at	4.54287854	1.12
	210471_s_at	-5.1127867	0.629
	210079_x_at	5.54624539	0.319
	231524_at	-6.8038966	0.105
	208213_s_at	-7.9657843	0.061
	203402_at	-3.0232698	3.72
	211791_s_at	-6.148161	0.211
	221413_at	-3.8975434	1.79
	207635_s_at	5.06371071	0.541
	210036_s_at	2.68965988	3.69
	205262_at	4.08804003	1.19
	223726_at	5.76415042	0.226
	220802_at	-7.6877995	0.0565
	1555304_a_at	3.83856373	1.55
	242502_at	5.19495524	0.801
	1555074_a_at	8.64963854	0.0343

	221023_s_at	4.50963525	0.788
	211046_at	5.31616883	0.547
	211045_s_at	6.35697504	0.163
	1555316_a_at	4.21357092	1.73
	224099_at	4.62237646	0.547
	1552742_at	5.29902769	0.502
	206231_at	-8.5381781	0.0287
	220116_at	6.09788782	0.29
	244040_at	4.10780329	1.26
	205903_s_at	4.92576861	0.648
	231103_at	5.33934515	0.472
	205902_at	-5.7878665	0.241
	204401_at	-7.3173188	0.0635
	204487_s_at	-3.4312869	3.55
	211217_s_at	-4.6583558	0.839
	211486_s_at	3.61528704	1.48
	210508_s_at	4.19759996	0.996
	205737_at	5.10283704	0.71
	228579_at	-5.2002495	0.396
	206573_at	5.67300254	0.23
	1557042_at	-6.9766453	0.114
	221083_at	3.68594159	1.42
	243209_at	7.55979192	0.0773
	223891_at	5.48035746	0.57
	244623_at	6.50635267	0.208
	208349_at	5.12784104	0.536
	217590_s_at	5.65835576	0.381
	205802_at	4.49005085	0.903
	211602_s_at	5.36884914	0.59
	205803_s_at	-5.7878665	0.251
	215288_at	5.41119543	0.447
	206425_s_at	-4.8038966	0.646
	210814_at	-7.615287	0.0931
	212059_s_at	-2.7369656	4.99
	224219_s_at	5.28208783	0.807
	220818_s_at	4.84476888	0.613
	224220_x_at	5.06854386	0.442
	220817_at	-7.6967511	0.103
	220552_at	-5.9126729	0.427
	206528_at	5.37482304	0.642
	217287_s_at	5.61528704	0.351

	208589_at	5.19495524	0.532
	234407_s_at	-5.8614476	0.406
	237069_s_at	4.08558856	2.21
	237070_at	4.76415042	1.01
	206479_at	5.15842936	0.775
	214410_at	8.64385619	0.0521
	205708_s_at	-4.127841	0.907
	239291_at	4.29902769	1.46
	1555252_a_at	-4.5769059	0.662
	239684_at	5.36884914	0.438
	1554722_at	5.58727266	0.401
	216452_at	-4.8868329	0.395
	233022_at	5.28208783	0.379
	211422_at	6.7239441	0.152
	220463_at	10.9400467	0.00568
	219360_s_at	6.48035746	0.13
	223935_at	4.28208783	0.83
	221102_s_at	-4.9839316	0.504
	224412_s_at	-4.011588	0.47
	234864_s_at	-5.4235262	0.353
	240389_at	5.11778738	0.305
	237884_x_at	4.28489736	1.49
	223323_x_at	3.86564761	1.47
	231689_at	5.36884914	0.322
	223324_s_at	5.65108776	0.124
	239484_at	8.1258247	0.0339
	243483_at	4.77595973	1.29
	220226_at	5.60121185	0.331
	219282_s_at	-4.2351443	0.907
	222855_s_at	-7.2216232	0.082
	1555291_at	5.82828076	0.327
	1552586_at	5.82828076	0.228
	219516_at	4.36884914	0.788
	208267_at	-6.9040081	0.107
	1555042_at	8.29675752	0.0419
	206827_s_at	4.13796526	0.955
	1559405_a_at	6.40506933	0.206
**Cytokines**	212659_s_at	-2.3959287	4.27
	212657_s_at	-2.2792838	4.06
	216243_s_at	-2.3658714	2.93
	216244_at	5.27648512	0.67

	207849_at	5.86144762	0.377
	217181_at	8.02843989	0.0353
	207906_at	-6.8800197	0.157
	205798_at	3.23786383	1.22
	226218_at	3.10780329	0.987
	204773_at	4.64746744	0.453
	1552646_at	-7.9407555	0.0482
	207375_s_at	-4.9930916	0.532
	206618_at	-2.4500844	2.22
	1561853_a_at	-4.908334	0.701
	1552912_a_at	-9.5435513	0.0209
	209821_at	4.35107444	2.01
	225669_at	-3.1328943	1.89
	204191_at	-3.3075728	1.6
	225661_at	-3.4723291	0.979
	236478_at	5.89539496	0.282
	227125_at	-4.7178568	0.398
	204786_s_at	-6.3335161	0.0825
	204785_x_at	8.26979047	0.0511
	207113_s_at	-4.046921	1.17
	207643_s_at	-2.6529013	3.83
	203508_at	-2.9105018	5
	202687_s_at	-3.2933589	1.21
	214329_x_at	-2.954557	1.2
	202688_at	-3.6082323	0.914
	210314_x_at	-2.8783214	2.94
	223502_s_at	-2.6438562	2.89
	223501_at	-3.1976	0.662
	221085_at	5.13289427	0.724
	229242_at	6.40506933	0.156
	204116_at	-3.2378638	1.37
	205291_at	-3.4787482	1.1
	207539_s_at	-5.7720125	0.326
	207538_at	-7.7547721	0.0645
	203233_at	-2.5063527	3.39
	211517_s_at	3.42662547	1.27
	210744_s_at	5.1896803	0.322
	211516_at	8.66712597	0.0395
	207902_at	-11.143666	0.00524
	205945_at	-2.8468432	2.2
	217489_s_at	-2.6803821	2.01

	226333_at	-3.6803821	1.28
	204912_at	-4.4932965	0.466
	209575_at	-3.2792838	1.65
	207901_at	4.97482942	0.967
	229101_at	-11.143666	5.3
	205707_at	-2.8468432	4.27
	228685_at	-2.6803821	3.85
	229295_at	-3.6803821	3.69
	219255_x_at	-4.4932965	1.23
	224361_s_at	-3.2792838	0.815
	224156_x_at	-5.1844246	0.51
	221926_s_at	-5.9301604	0.233
	64440_at	8.62095579	0.0334
	221947_at	2.9770996	2.35
	224514_x_at	3.36884914	1.94
	227997_at	4.58380881	0.955
	229263_at	4.96128289	0.81
	1552995_at	-5.2216232	0.357
	211676_s_at	-2.5734669	1.61
	202727_s_at	-2.9323613	1.4
	242903_at	4.86564761	0.194
	201642_at	-2.7369656	3.67
	210354_at	5.42973138	0.378
	205067_at	-3.3306103	1.54
	39402_at	-3.5129253	1.38
	210401_at	-2.1392358	5.6
**Receptors**	204088_at	-3.1584294	2.34
	230741_at	-3.3703403	0.64
	207091_at	-4.615287	0.573
	1560874_at	4.66566056	0.646
	223955_at	8.10979459	0.0477
	228752_at	6.69077724	0.0825
	227429_at	4.83650127	0.693
	212090_at	-3.7898605	0.764
	233892_at	5.17397021	0.376
	233220_at	5.94786238	0.243
	233171_at	9.43971547	0.0209
	221140_s_at	-3.3466648	2.12
	1562412_at	-4.7408179	0.648
	225463_x_at	4.3047188	0.583
	222140_s_at	-4.3540217	0.359

	220642_x_at	4.96128289	0.321
	204137_at	-4.18705	0.428
	221966_at	-5.2216232	0.344
	219430_at	5.68038207	0.301
	43934_at	-6.3928946	0.183
	242592_at	6.23242994	0.308
	223620_at	-5.5328249	0.166
	1552440_at	5.15328606	0.585
	214506_at	-5.3335161	0.327
	219898_at	7.84808924	0.0958
	234303_s_at	4.86144762	0.658
	205056_s_at	5.86144762	0.197
	244877_at	-5.9126729	0.195
	1556039_s_at	-7.3265521	0.114
	1556038_at	4.54962012	0.785
	221299_at	4.86144762	0.614
	231745_at	-7.0724221	0.11
	237559_at	5.09295553	0.562
	1553063_at	4.48035746	0.602
	220265_at	6.58727266	0.24
	220264_s_at	-7.1218004	0.116
	211977_at	-2.8059129	3.97
	211979_at	-3.5649048	2
	221140_s_at	-3.3466648	2.12
	223887_at	-4.0303245	1.1
	206696_at	7.00786969	0.0921
	227970_at	7.60682546	0.061
	220901_at	8.88172002	0.0259
	223423_at	-3.5838088	0.447
	230369_at	5.42352623	0.406
	232350_x_at	-7.0802099	0.0867
	214104_at	-8.7208972	0.0255
	235961_at	3.87620139	1.11
	206972_s_at	4.66200354	0.863
	206971_at	4.50635267	0.833
	207183_at	-12.000831	0.00261
	222140_s_at	-4.3540217	0.359
	220642_x_at	4.96128289	0.321
	225463_x_at	4.3047188	0.583
	223531_x_at	4.42352623	0.533
	208524_at	4.83650127	0.577

	231166_at	6.1896803	0.0857
	244509_at	-7.0435864	0.0835
	239533_at	-3.2243173	1.04
	210264_at	-4.7918574	0.654
	207055_at	-5.5530028	0.239
	1556155_at	4.18442457	1.29
	211266_s_at	5.14305414	0.349
	206236_at	5.94786238	0.246
	1553317_s_at	-6.6012119	0.134
	244434_at	4.17136842	0.893
	1553316_at	5.19495524	0.492
	223767_at	-4.1976	0.539
	214467_at	-3.72547	0.421
	212444_at	6.12784104	0.204
	203108_at	6.40506933	0.178
	235563_at	4.76023537	0.764
	220481_at	-4.8323852	0.896
	206488_s_at	-3.2378638	0.775
	242197_x_at	3.44065503	0.614
	228766_at	-3.7958593	0.366
	209555_s_at	5.67300254	0.0921
	209554_at	3.77793738	2.21
	226531_at	-3.4297314	1.33
	218811_at	5.73312353	0.207
	231406_at	-2.4739312	4.88
	218812_s_at	-2.5395195	3.07
	1558426_x_at	-3.046921	2.53
	230347_at	3.45482237	1.62
	217529_at	-3.1713684	1.43
	221864_at	-2.8365013	4.62
	202764_at	-3.1328943	3.48
	1557477_at	-3.3928946	0.883
	225250_at	4.06371071	1.07
	234140_s_at	-8.2653446	0.0218
	225246_at	-9.1177874	0.0106
	210176_at	-3.2108968	1.22
	204924_at	-3.6420539	1.07
	206271_at	-5.21625	0.662
	239587_at	-5.9839316	0.328
	242667_at	4.76807613	1.12
	232068_s_at	-1.6989977	3.14

	224341_x_at	-2.7858752	1.53
	221060_s_at	-2.7466158	1.55
	1552798_a_at	-3.5411981	1.07
	210166_at	-3.6420539	0.967
	239021_at	-2.3364277	2.56
	207446_at	-4.3959287	0.583
	222952_s_at	-4.8365013	0.506
	220146_at	-4.6583558	0.427
	220832_at	-3.2933589	0.921
	229560_at	-3.7958593	0.71
	223903_at	-5.8783214	0.234
	223751_x_at	9.09394064	0.0168
	223750_s_at	-10.082946	0.00966

Supplemental Table S4. Data used for the elaboration of graph 1B, data obtained from the GSE99039 database.

ID GEN	Log 2 Fold Change	Log 10 *P*-value
**210401_at**	-2.1392358	5.6
**229101_at**	-11.143666	5.3
**203508_at**	-2.9105018	5
**212059_s_at**	-2.7369656	4.99
**231406_at**	-2.4739312	4.88
**221864_at**	-2.8365013	4.62
**205707_at**	-2.8468432	4.27
**212659_s_at**	-2.3959287	4.27
**212657_s_at**	-2.2792838	4.06
**211977_at**	-2.8059129	3.97
**228685_at**	-2.6803821	3.85
**207643_s_at**	-2.6529013	3.83
**203402_at**	-3.0232698	3.72
**210036_s_at**	2.68965988	3.69
**229295_at**	-3.6803821	3.69
**201642_at**	-2.7369656	3.67
**204487_s_at**	-3.4312869	3.55
**202764_at**	-3.1328943	3.48
**203233_at**	-2.5063527	3.39
**243334_at**	-3.1584294	3.22
**232068_s_at**	-1.6989977	3.14
**218812_s_at**	-2.5395195	3.07
**208432_s_at**	3.14560532	2.97
**205148_s_at**	-2.6438562	2.95
**210314_x_at**	-2.8783214	2.94
**216243_s_at**	-2.3658714	2.93
**211806_s_at**	-2.4579896	2.92
**223502_s_at**	-2.6438562	2.89
**213499_at**	3.29335894	2.73
**1555230_a_at**	3.33642766	2.57
**239021_at**	-2.3364277	2.56
**1558426_x_at**	-3.046921	2.53
**221947_at**	2.9770996	2.35
**232936_at**	3.77004299	2.34
**204088_at**	-3.1584294	2.34
**210119_at**	-2.6170561	2.26
**206618_at**	-2.4500844	2.22
**237069_s_at**	4.08558856	2.21

**209554_at**	3.77793738	2.21
**211304_x_at**	3.18442457	2.2
**205945_at**	-2.8468432	2.2
**221140_s_at**	-3.3466648	2.12
**221140_s_at**	-3.3466648	2.12
**222289_at**	3.53115606	2.09
**234756_at**	3.36141638	2.04
**238428_at**	-2.9323613	2.03
**222960_at**	3.3075728	2.02
**209821_at**	4.35107444	2.01
**217489_s_at**	-2.6803821	2.01
**211979_at**	-3.5649048	2
**208299_at**	3.13289427	1.98
**224514_x_at**	3.36884914	1.94
**234750_at**	3.55131045	1.9
**225669_at**	-3.1328943	1.89
**222379_at**	4.07096652	1.88
**222922_at**	-2.7661119	1.84
**230585_at**	-2.3437325	1.83
**1552507_at**	3.77004299	1.81
**221413_at**	-3.8975434	1.79
**224137_at**	4.0540927	1.75
**229199_at**	4.65108776	1.74
**1555316_a_at**	4.21357092	1.73
**214495_at**	3.6384562	1.72
**224291_at**	3.89969509	1.72
**236407_at**	-3.7858752	1.7
**209575_at**	-3.2792838	1.65
**231774_at**	3.45640514	1.64
**211830_s_at**	3.74661576	1.64
**230347_at**	3.45482237	1.62
**211676_s_at**	-2.5734669	1.61
**204191_at**	-3.3075728	1.6
**1555304_a_at**	3.83856373	1.55
**221060_s_at**	-2.7466158	1.55
**205067_at**	-3.3306103	1.54
**224341_x_at**	-2.7858752	1.53
**206765_at**	-2.8889687	1.52
**207237_at**	2.94341647	1.52
**237884_x_at**	4.28489736	1.49
**227647_at**	-3.3219281	1.49

**211486_s_at**	3.61528704	1.48
**205149_s_at**	-3.9885044	1.48
**207162_s_at**	3.38232537	1.48
**223323_x_at**	3.86564761	1.47
**211802_x_at**	3.83856373	1.46
**239291_at**	4.29902769	1.46
**217529_at**	-3.1713684	1.43
**221083_at**	3.68594159	1.42
**217515_s_at**	3.73312353	1.41
**202727_s_at**	-2.9323613	1.4
**211314_at**	3.91267295	1.4
**206996_x_at**	-3.917025	1.39
**39402_at**	-3.5129253	1.38
**204116_at**	-3.2378638	1.37
**226531_at**	-3.4297314	1.33
**210263_at**	3.56149422	1.32
**211451_s_at**	3.55300276	1.31
**206842_at**	-4.2002495	1.3
**1556155_at**	4.18442457	1.29
**243483_at**	4.77595973	1.29
**226333_at**	-3.6803821	1.28
**211517_s_at**	3.42662547	1.27
**244040_at**	4.10780329	1.26
**1555993_at**	-4.1844246	1.25
**228581_at**	4.36289988	1.23
**219255_x_at**	-4.4932965	1.23
**210176_at**	-3.2108968	1.22
**205798_at**	3.23786383	1.22
**202687_s_at**	-3.2933589	1.21
**211315_s_at**	4.36289988	1.2
**221631_at**	3.82623293	1.2
**214329_x_at**	-2.954557	1.2
**205262_at**	4.08804003	1.19
**207693_at**	-3.5977144	1.18
**207113_s_at**	-4.046921	1.17
**244099_at**	4.16617886	1.13
**242667_at**	4.76807613	1.12
**210078_s_at**	4.54287854	1.12
**235961_at**	3.87620139	1.11
**205291_at**	-3.4787482	1.1
**223887_at**	-4.0303245	1.1

**1554748_at**	4.08314124	1.08
**204924_at**	-3.6420539	1.07
**225250_at**	4.06371071	1.07
**1552798_a_at**	-3.5411981	1.07
**1552508_at**	4.46434514	1.05
**239533_at**	-3.2243173	1.04
**237070_at**	4.76415042	1.01
**210179_at**	4.50307753	1.01
**201733_at**	3.66383089	0.996
**214769_at**	-3.8180706	0.996
**210508_s_at**	4.19759996	0.996
**226218_at**	3.10780329	0.987
**225661_at**	-3.4723291	0.979
**213832_at**	4.56661319	0.967
**207901_at**	4.97482942	0.967
**210166_at**	-3.6420539	0.967
**236013_at**	-4.1379653	0.963
**213714_at**	4.84892053	0.955
**206827_s_at**	4.13796526	0.955
**227997_at**	4.58380881	0.955
**222923_s_at**	-3.7350433	0.924
**220832_at**	-3.2933589	0.921
**230849_at**	4.72165834	0.917
**202688_at**	-3.6082323	0.914
**205708_s_at**	-4.127841	0.907
**219282_s_at**	-4.2351443	0.907
**232411_at**	5.20024954	0.903
**232411_at**	5.20024954	0.903
**205802_at**	4.49005085	0.903
**208020_s_at**	4.36289988	0.9
**244113_at**	4.44850859	0.896
**220481_at**	-4.8323852	0.896
**244434_at**	4.17136842	0.893
**1557477_at**	-3.3928946	0.883
**232127_at**	3.90400809	0.873
**206972_s_at**	4.66200354	0.863
**211217_s_at**	-4.6583558	0.839
**210363_s_at**	4.53616832	0.833
**210363_s_at**	4.53616832	0.833
**206971_at**	4.50635267	0.833
**223935_at**	4.28208783	0.83

**224361_s_at**	-3.2792838	0.815
**229263_at**	4.96128289	0.81
**224219_s_at**	5.28208783	0.807
**242502_at**	5.19495524	0.801
**221023_s_at**	4.50963525	0.788
**219516_at**	4.36884914	0.788
**215365_at**	4.83238516	0.785
**1556038_at**	4.54962012	0.785
**206479_at**	5.15842936	0.775
**206488_s_at**	-3.2378638	0.775
**206950_at**	5.53951953	0.764
**212090_at**	-3.7898605	0.764
**235563_at**	4.76023537	0.764
**221961_at**	4.1896803	0.757
**226273_at**	4.46434514	0.747
**221085_at**	5.13289427	0.724
**244256_at**	4.86564761	0.719
**210383_at**	4.96578428	0.717
**205737_at**	5.10283704	0.71
**229560_at**	-3.7958593	0.71
**207103_at**	-5.3393451	0.703
**1561853_a_at**	-4.908334	0.701
**227429_at**	4.83650127	0.693
**216244_at**	5.27648512	0.67
**1555252_a_at**	-4.5769059	0.662
**223501_at**	-3.1976	0.662
**206271_at**	-5.21625	0.662
**234303_s_at**	4.86144762	0.658
**210264_at**	-4.7918574	0.654
**240614_at**	4.83238516	0.652
**1552863_a_at**	-4.3898669	0.65
**208560_at**	4.82828076	0.648
**205903_s_at**	4.92576861	0.648
**1562412_at**	-4.7408179	0.648
**206425_s_at**	-4.8038966	0.646
**1560874_at**	4.66566056	0.646
**206528_at**	5.37482304	0.642
**230741_at**	-3.3703403	0.64
**206384_at**	-4.8698599	0.636
**217556_at**	-5.2378638	0.629
**210471_s_at**	-5.1127867	0.629

**233059_at**	5.17397021	0.614
**221299_at**	4.86144762	0.614
**242197_x_at**	3.44065503	0.614
**220818_s_at**	4.84476888	0.613
**208477_at**	-5.1844246	0.611
**1553063_at**	4.48035746	0.602
**211592_s_at**	5.49980982	0.6
**211602_s_at**	5.36884914	0.59
**38069_at**	5.0540927	0.588
**1552440_at**	5.15328606	0.585
**225463_x_at**	4.3047188	0.583
**225463_x_at**	4.3047188	0.583
**207446_at**	-4.3959287	0.583
**210967_x_at**	-4.7331235	0.58
**207141_s_at**	-4.8282808	0.578
**208524_at**	4.83650127	0.577
**207091_at**	-4.615287	0.573
**223891_at**	5.48035746	0.57
**237559_at**	5.09295553	0.562
**208251_at**	4.94786238	0.547
**211046_at**	5.31616883	0.547
**224099_at**	4.62237646	0.547
**204722_at**	-4.7027499	0.545
**220010_at**	-5.0493076	0.545
**207635_s_at**	5.06371071	0.541
**223767_at**	-4.1976	0.539
**208349_at**	5.12784104	0.536
**223531_x_at**	4.42352623	0.533
**208589_at**	5.19495524	0.532
**207375_s_at**	-4.9930916	0.532
**208397_x_at**	4.94786238	0.521
**221095_s_at**	5.0540927	0.516
**224156_x_at**	-5.1844246	0.51
**222952_s_at**	-4.8365013	0.506
**221102_s_at**	-4.9839316	0.504
**1552742_at**	5.29902769	0.502
**224528_s_at**	5.50635267	0.496
**1553316_at**	5.19495524	0.492
**228504_at**	5.71028355	0.478
**207600_at**	4.95232202	0.472
**231103_at**	5.33934515	0.472

**224412_s_at**	-4.011588	0.47
**204912_at**	-4.4932965	0.466
**209235_at**	-4.8119789	0.455
**204773_at**	4.64746744	0.453
**215288_at**	5.41119543	0.447
**223423_at**	-3.5838088	0.447
**224220_x_at**	5.06854386	0.442
**211301_at**	5.12784104	0.438
**239684_at**	5.36884914	0.438
**231740_at**	5.29335894	0.437
**238636_at**	5.18442457	0.434
**204137_at**	-4.18705	0.428
**220552_at**	-5.9126729	0.427
**220146_at**	-4.6583558	0.427
**214467_at**	-3.72547	0.421
**207142_at**	5.65108776	0.42
**201735_s_at**	-4.4739312	0.411
**221401_at**	-5.5872727	0.411
**208578_at**	5.27090409	0.41
**234407_s_at**	-5.8614476	0.406
**230369_at**	5.42352623	0.406
**205304_s_at**	-5.4297314	0.402
**1554722_at**	5.58727266	0.401
**210364_at**	5.20556334	0.399
**206692_at**	-5.2933589	0.399
**227125_at**	-4.7178568	0.398
**228579_at**	-5.2002495	0.396
**216452_at**	-4.8868329	0.395
**210853_at**	5.09295553	0.393
**230531_at**	-4.9657843	0.393
**211427_s_at**	6.23242994	0.389
**217590_s_at**	5.65835576	0.381
**233022_at**	5.28208783	0.379
**210354_at**	5.42973138	0.378
**206381_at**	5.41734766	0.377
**207849_at**	5.86144762	0.377
**233892_at**	5.17397021	0.376
**236359_at**	-6.0023102	0.373
**224530_s_at**	-5.5803532	0.367
**228766_at**	-3.7958593	0.366
**219564_at**	5.79585928	0.364

**207413_s_at**	5.26534457	0.363
**207413_s_at**	5.26534457	0.363
**222140_s_at**	-4.3540217	0.359
**222140_s_at**	-4.3540217	0.359
**1552995_at**	-5.2216232	0.357
**234864_s_at**	-5.4235262	0.353
**203950_s_at**	-5.5195281	0.352
**217287_s_at**	5.61528704	0.351
**239464_at**	-4.7216583	0.35
**211266_s_at**	5.14305414	0.349
**221966_at**	-5.2216232	0.344
**208514_at**	5.27090409	0.343
**210108_at**	6.07825901	0.335
**207248_at**	5.87832144	0.333
**220226_at**	5.60121185	0.331
**239587_at**	-5.9839316	0.328
**1555291_at**	5.82828076	0.327
**214506_at**	-5.3335161	0.327
**220791_x_at**	5.84476888	0.326
**220791_x_at**	5.84476888	0.326
**207539_s_at**	-5.7720125	0.326
**210403_s_at**	-5.7178568	0.324
**231689_at**	5.36884914	0.322
**210744_s_at**	5.1896803	0.322
**242973_at**	5.76415042	0.321
**220642_x_at**	4.96128289	0.321
**220642_x_at**	4.96128289	0.321
**210079_x_at**	5.54624539	0.319
**228269_x_at**	5.55979192	0.308
**242592_at**	6.23242994	0.308
**244708_at**	5.94786238	0.307
**240389_at**	5.11778738	0.305
**219430_at**	5.68038207	0.301
**1552602_at**	-6.078259	0.298
**215014_at**	6.42973138	0.298
**207527_at**	5.82828076	0.296
**220116_at**	6.09788782	0.29
**201734_at**	-4.5496201	0.289
**201732_s_at**	5.13796526	0.284
**238780_s_at**	-5.5597919	0.282
**236478_at**	5.89539496	0.282

**205508_at**	-5.2598064	0.279
**210185_at**	5.54624539	0.255
**205803_s_at**	-5.7878665	0.251
**211817_s_at**	-5.7485536	0.248
**235781_at**	-5.9213902	0.246
**206236_at**	5.94786238	0.246
**205845_at**	-5.6012119	0.243
**233220_at**	5.94786238	0.243
**205902_at**	-5.7878665	0.241
**220265_at**	6.58727266	0.24
**207055_at**	-5.5530028	0.239
**62987_r_at**	5.71785677	0.238
**223903_at**	-5.8783214	0.234
**1569355_at**	6.13796526	0.234
**221926_s_at**	-5.9301604	0.233
**1555694_a_at**	-5.9657843	0.23
**206573_at**	5.67300254	0.23
**220776_at**	5.73312353	0.228
**1552586_at**	5.82828076	0.228
**204723_at**	-6.148161	0.228
**233688_at**	6.34519787	0.226
**223726_at**	5.76415042	0.226
**209530_at**	-5.9839316	0.223
**221321_s_at**	6.03032454	0.223
**243244_at**	6.33351607	0.221
**206612_at**	5.96578428	0.218
**238747_at**	5.9567955	0.217
**239118_at**	-6.5195281	0.211
**211791_s_at**	-6.148161	0.211
**205303_at**	-6.2653446	0.21
**229057_at**	6.82173792	0.209
**244623_at**	6.50635267	0.208
**231737_at**	-6.4932965	0.207
**218811_at**	5.73312353	0.207
**1559405_a_at**	6.40506933	0.206
**235225_at**	6.41734766	0.204
**212444_at**	6.12784104	0.204
**207776_s_at**	6.32192809	0.202
**205056_s_at**	5.86144762	0.197
**221307_at**	6.09788782	0.195
**244877_at**	-5.9126729	0.195

**242903_at**	4.86564761	0.194
**43934_at**	-6.3928946	0.183
**206762_at**	6.07825901	0.181
**211827_s_at**	6.67300254	0.18
**203108_at**	6.40506933	0.178
**206704_at**	6.78627323	0.175
**206981_at**	6.41734766	0.173
**1553347_s_at**	6.41734766	0.17
**223620_at**	-5.5328249	0.166
**211045_s_at**	6.35697504	0.163
**237186_at**	-6.3335161	0.161
**207906_at**	-6.8800197	0.157
**229242_at**	6.40506933	0.156
**211422_at**	6.7239441	0.152
**210402_at**	-6.5872727	0.144
**228436_at**	6.64963854	0.141
**1555098_a_at**	-6.9407555	0.138
**210380_s_at**	6.79265685	0.137
**230547_at**	-7.133907	0.136
**1553317_s_at**	-6.6012119	0.134
**219360_s_at**	6.48035746	0.13
**223324_s_at**	5.65108776	0.124
**220264_s_at**	-7.1218004	0.116
**208479_at**	6.75789143	0.114
**208479_at**	6.75789143	0.114
**1557042_at**	-6.9766453	0.114
**1556039_s_at**	-7.3265521	0.114
**231745_at**	-7.0724221	0.11
**221585_at**	-7.011588	0.108
**235467_s_at**	-7.2944909	0.107
**208267_at**	-6.9040081	0.107
**231524_at**	-6.8038966	0.105
**220817_at**	-7.6967511	0.103
**236783_at**	-6.9284021	0.103
**219898_at**	7.84808924	0.0958
**210814_at**	-7.615287	0.0931
**206696_at**	7.00786969	0.0921
**209555_s_at**	5.67300254	0.0921
**1555246_a_at**	7.35225263	0.0904
**232350_x_at**	-7.0802099	0.0867
**34726_at**	7.58450091	0.0862

**231166_at**	6.1896803	0.0857
**244509_at**	-7.0435864	0.0835
**204786_s_at**	-6.3335161	0.0825
**228752_at**	6.69077724	0.0825
**222855_s_at**	-7.2216232	0.082
**243209_at**	7.55979192	0.0773
**232512_at**	-7.3928946	0.0726
**223727_at**	-7.4447335	0.0711
**207998_s_at**	-7.3953214	0.0701
**207538_at**	-7.7547721	0.0645
**204401_at**	-7.3173188	0.0635
**208213_s_at**	-7.9657843	0.061
**227970_at**	7.60682546	0.061
**232128_s_at**	8.05505162	0.057
**220802_at**	-7.6877995	0.0565
**208404_x_at**	-7.8152246	0.0545
**214410_at**	8.64385619	0.0521
**204785_x_at**	8.26979047	0.0511
**222901_s_at**	-8.1750122	0.0506
**1552646_at**	-7.9407555	0.0482
**223955_at**	8.10979459	0.0477
**1554749_s_at**	-8.0359933	0.0472
**1559420_x_at**	-7.7239441	0.0472
**1555042_at**	8.29675752	0.0419
**211516_at**	8.66712597	0.0395
**226274_at**	8.09788782	0.0386
**217181_at**	8.02843989	0.0353
**1555074_a_at**	8.64963854	0.0343
**239484_at**	8.1258247	0.0339
**205985_x_at**	8.17918792	0.0334
**64440_at**	8.62095579	0.0334
**207869_s_at**	9.30585973	0.032
**240650_at**	-8.4397155	0.0306
**206231_at**	-8.5381781	0.0287
**208377_s_at**	-8.5221776	0.0264
**220901_at**	8.88172002	0.0259
**214104_at**	-8.7208972	0.0255
**234140_s_at**	-8.2653446	0.0218
**1552912_a_at**	-9.5435513	0.0209
**233171_at**	9.43971547	0.0209
**1559419_at**	-8.9372151	0.0195

**223751_x_at**	9.09394064	0.0168
**242410_s_at**	-10.431723	0.0132
**225246_at**	-9.1177874	0.0106
**223750_s_at**	-10.082946	0.00966
**208359_s_at**	10.6186856	0.00789
**231355_at**	10.5674339	0.00656
**220463_at**	10.9400467	0.00568
**207902_at**	-11.143666	0.00524
**243893_at**	-11.480357	0.0048
**208437_at**	-11.565246	0.00349
**231066_s_at**	-11.650798	0.00349
**207183_at**	-12.000831	0.00261

Supplemental Table S5. Data used for the elaboration of figure 2, data obtained from SHINY 8.1 software.

Enrichment FDR	nGenes	Fold Enrichment	Pathway	Genes
3.41E-15	13	32.6369322	Path:hsa04060 Cytokine-cytokine receptor interaction	IL17RA IL1B IL2RA IL2RG IL4R IL10RB IL12B IL12RB1 TNFRSF1A TNFRSF1B TNFSF13 IL18R1 IL33
1.47E-08	6	68.1320099	Path:hsa05321 Inflammatory bowel disease	IL1B IL2RG IL4R IL12B IL12RB1 TLR2
1.29E-07	6	44.7331378	Path:hsa04061 Viral protein interaction with cytokine and cytokine receptor	IL2RA IL2RG IL10RB TNFRSF1A TNFRSF1B IL18R1
1.86E-06	6	27.3369176	Path:hsa04630 JAK-STAT signaling pathway	IL2RA IL2RG IL4R IL10RB IL12B IL12RB1
2.50E-06	5	40.1139551	Path:hsa04658 Th1 and Th2 cell differentiation	IL2RA IL2RG IL4R IL12B IL12RB1
2.50E-06	6	24.740674	Path:hsa05152 Tuberculosis	IL1B IL10RB IL12B TLR1 TLR2 TNFRSF1A
4.79E-06	5	34.171147	Path:hsa04659 Th17 cell differentiation	IL1B IL2RA IL2RG IL4R IL12RB1
9.43E-06	6	18.4524194	Path:hsa04020 Calcium signaling pathway	P2RX1 P2RX4 CACNA1E CACNA1S ORAI1 ORAI3
1.31E-05	5	26.5502437	Path:hsa05162 Measles	IL1B IL2RA IL2RG IL12B TLR2
0.0001029	4	28.6639524	Path:hsa04620 Toll-like receptor signaling pathway	IL1B IL12B TLR1 TLR2
0.0001029	4	29.2315554	Path:hsa05142 Chagas disease	IL1B IL12B TLR2 TNFRSF1A
0.00012127	4	26.3605991	Path:hsa04668 TNF signaling pathway	IL1B TNFRSF1A TNFRSF1B IL18R1
0.00012127	4	26.3605991	Path:hsa05145 Toxoplasmosis	IL10RB IL12B TLR2 TNFRSF1A
0.00027833	4	20.9389156	Path:hsa04936 Alcoholic liver disease	IL17RA IL1B IL12B TNFRSF1A

0.0002974	5	12.5526662	Path:hsa04010 MAPK signaling pathway	IL1B CACNG8 TNFRSF1A CACNA1E CACNA1S
0.00039971	3	38.8471986	Path:hsa05134 Legionellosis	IL1B IL12B TLR2
0.00048567	4	17.2654216	Path:hsa05164 Influenza A	IL1B IL12B TNFRSF1A IL33
0.0008389	3	29.135399	Path:hsa05140 Leishmaniasis	IL1B IL12B TLR2
0.00103082	4	13.7962014	Path:hsa05417 Lipid and atherosclerosis	IL1B IL12B TLR2 TNFRSF1A
0.00126938	4	12.7258065	Path:hsa05171 Coronavirus disease-COVID-19	IL1B IL12B TLR2 TNFRSF1A
0.00126938	3	24.0683731	Path:hsa05323 Rheumatoid arthritis	IL1B TLR2 TNFSF13
0.00149946	3	22.3665689	Path:hsa04640 Hematopoietic cell lineage	IL1B IL2RA IL4R
0.00156575	3	21.7087287	Path:hsa05146 Amoebiasis	IL1B IL12B TLR2
0.00176222	5	7.75311738	Path:hsa05022 Pathways of neurodegeneration-multiple diseases	IL1B SNCA TNFRSF1A TNFRSF1B CACNA1S
0.00275611	5	6.96317712	Path:hsa05200 Pathways in cancer	IL2RA IL2RG IL4R IL12B IL12RB1
0.00450629	2	39.8971229	Path:hsa05143 African trypanosomiasis	IL1B IL12B
0.00451266	4	8.34007654	Path:hsa04151 PI3K-Akt signaling pathway	IL2RA IL2RG IL4R TLR2
0.00451266	3	13.9263542	Path:hsa04217 Necroptosis	IL1B TNFRSF1A IL33
0.00451266	2	38.8471986	Path:hsa05340 Primary immunodeficiency	IL2RG ORAI1
0.00526851	2	34.3300825	Path:hsa04940 Type I diabetes mellitus	IL1B IL12B
0.0056499	4	7.68850806	Path:hsa05010 Alzheimer disease	IL1B SNCA TNFRSF1A CACNA1S

0.00640004	2	30.1263989	Path:hsa05144 Malaria	IL1B TLR2
0.00876103	3	10.5442396	Path:hsa05170 Human immunodeficiency virus 1 infection	TLR2 TNFRSF1A TNFRSF1B
0.00963151	2	23.4316436	Path:hsa04623 Cytosolic DNA-sensing pathway	IL1B IL33
0.00963151	3	9.88522465	Path:hsa05163 Human cytomegalovirus infection	IL1B IL10RB TNFRSF1A
0.00963151	3	9.97428073	Path:hsa05166 Human T-cell leukemia virus 1 infection	IL2RA IL2RG TNFRSF1A
0.00965909	2	22.71067	Path:hsa04927 Cortisol synthesis and secretion	CACNA1S ORAI1
0.01030093	2	21.3941094	Path:hsa04920 Adipocytokine signaling pathway	TNFRSF1A TNFRSF1B
0.01030093	2	21.3941094	Path:hsa04924 Renin secretion	CACNA1S ORAI1
0.01092817	4	5.96441838	Path:hsa05168 Herpes simplex virus 1 infection	IL1B IL12B TLR2 TNFRSF1A
0.01136975	3	8.89273222	Path:hsa05132 Salmonella infection	IL1B TLR2 TNFRSF1A
0.01155186	2	19.4235993	Path:hsa05133 Pertussis	IL1B IL12B
0.01157437	2	19.1713448	Path:hsa05412 Arrhythmogenic right ventricular cardiomyopathy	CACNG8 CACNA1S
0.01434091	2	16.9677419	Path:hsa04260 Cardiac muscle contraction	CACNG8 CACNA1S
0.01497398	2	16.4021505	Path:hsa05410 Hypertrophic cardiomyopathy	CACNG8 CACNA1S
0.0156076	2	15.8730489	Path:hsa04657 IL-17 signaling pathway	IL17RA IL1B

0.0162415	2	15.3770161	Path:hsa05414 Dilated cardiomyopathy	CACNG8 CACNA1S
0.01654842	2	15.0631995	Path:hsa04925 Aldosterone synthesis and secretion	CACNA1S ORAI1
0.01781205	2	14.1941687	Path:hsa04064 NF-kappa B signaling pathway	IL1B TNFRSF1A
0.01781205	2	14.1941687	Path:hsa04625 C-type lectin receptor signaling pathway	IL1B IL12B
0.02445053	2	11.9047867	Path:hsa04611 Platelet activation	P2RX1 ORAI1
0.02472187	2	11.7158218	Path:hsa04380 Osteoclast differentiation	IL1B TNFRSF1A
0.02882471	2	10.6970547	Path:hsa05418 Fluid shear stress and atherosclerosis	IL1B TNFRSF1A
0.03269638	2	9.90733925	Path:hsa04261 Adrenergic signaling in cardiomyocytes	CACNG8 CACNA1S
0.03336145	2	9.58567239	Path:hsa04921 Oxytocin signaling pathway	CACNG8 CACNA1S
0.03336145	2	9.52382934	Path:hsa04932 Non-alcoholic fatty liver disease	IL1B TNFRSF1A
0.03336145	2	9.52382934	Path:hsa04934 Cushing syndrome	CACNA1S ORAI1
0.05120877	2	7.49336827	Path:hsa05130 Pathogenic Escherichia coli infection	IL1B TNFRSF1A
0.05271537	2	7.30788885	Path:hsa05205 Proteoglycans in cancer	IL12B TLR2
0.06109998	2	6.67960882	Path:hsa04024 cAMP signaling pathway	CACNA1S ORAI1
0.06668691	1	24.6032258	Path:hsa01523 Antifolate resistance	IL1B

0.06989419	1	23.0655242	Path:hsa04215 Apoptosis-multiple species	TNFRSF1A
0.0706516	2	6.00078678	Path:hsa05131 Shigellosis	IL1B TNFRSF1A
0.07210977	2	5.8812492	Path:hsa04144 Endocytosis	IL2RA IL2RG
0.07885968	1	19.4235993	Path:hsa05330 Allograft rejection	IL12B
0.08125171	2	5.40730238	Path:hsa05020 Prion disease	IL1B CACNA1S
0.08433898	1	17.5737327	Path:hsa05332 Graft-versus-host disease	IL1B
0.09077635	1	16.045582	Path:hsa04930 Type II diabetes mellitus	CACNA1E
0.09322905	1	15.3770161	Path:hsa04672 Intestinal immune network for IgA production	TNFSF13
0.11398	1	12.3016129	Path:hsa04978 Mineral absorption	CLCN2
0.11955588	1	11.5327621	Path:hsa04929 GnRH secretion	CACNA1S
0.12126744	2	4.07788273	Path:hsa04080 Neuroactive ligand-receptor interaction	P2RX1 P2RX4
0.12126744	2	4.05547678	Path:hsa05014 Amyotrophic lateral sclerosis	TNFRSF1A TNFRSF1B
0.12497642	1	10.5442396	Path:hsa04622 RIG-I-like receptor signaling pathway	IL12B
0.13336089	1	9.71179966	Path:hsa04971 Gastric acid secretion	KCNJ15
0.14796399	1	8.58252063	Path:hsa04911 Insulin secretion	CACNA1S
0.14891078	1	8.29322218	Path:hsa04727 GABAergic synapse	CACNA1S
0.14891078	1	8.29322218	Path:hsa05235 PD-L1 expression and	TLR2

			PD-1 checkpoint pathway in cancer	
0.15323811	1	7.93652445	Path:hsa04912 GnRH signaling pathway	CACNA1S
0.15894559	1	7.53159974	Path:hsa04750 Inflammatory mediator regulation of TRP channels	IL1B
0.15998098	1	7.38096774	Path:hsa04933 AGE-RAGE signaling pathway in diabetic complications	IL1B
0.16979674	1	6.83422939	Path:hsa04931 Insulin resistance	TNFRSF1A
0.17287181	1	6.53182986	Path:hsa04725 Cholinergic synapse	CACNA1S
0.17287181	1	6.59014977	Path:hsa04726 Serotonergic synapse	CACNA1S
0.17850809	1	6.2024939	Path:hsa04071 Sphingolipid signaling pathway	TNFRSF1A
0.17850809	1	6.15080645	Path:hsa04935 Growth hormone synthesis secretion and action	CACNA1S
0.19479661	1	5.42718216	Path:hsa04210 Apoptosis	TNFRSF1A
0.19479661	1	5.50818488	Path:hsa04270 Vascular smooth muscle contraction	CACNA1S
0.19479661	1	5.38756769	Path:hsa05135 Yersinia infection	IL1B

Supplementary Figure S1. End point PCR, evaluation of neutrophil enrichment
1: Non Template Control (NTC),
2: Positive Control,
3: Healthy control,
4: Parkinson's Disease.

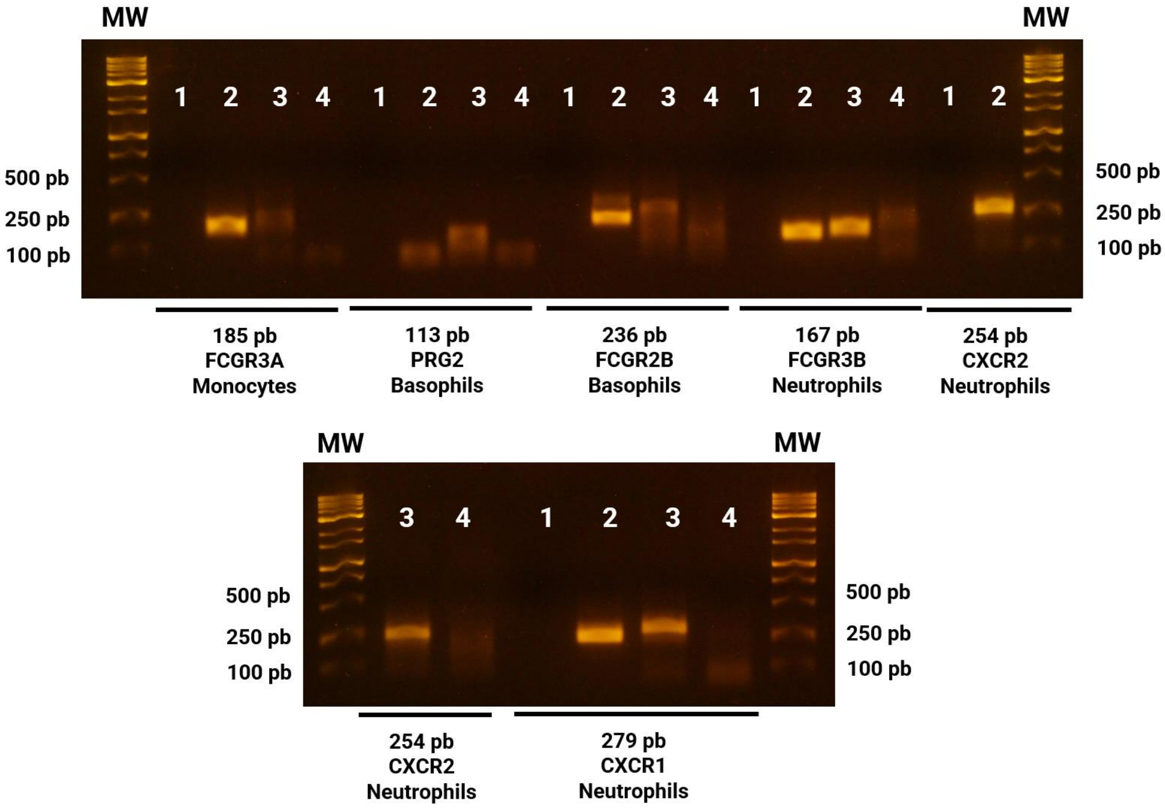

Supplement: Supplementary file 1 [file cimb-47-00459-s001.zip › cimb-3659082-supplementary.pdf]
